# Deaths from Ether

**Published:** 1881-12

**Authors:** 


### DEATHS FROM ETHER.

Two succeeding numbers of the *London Lancet* report each, a case of death on the table from ether. The anæsthetic mixture used in the Vienna General Hospital, is composed of alcohol, 90 parts; ether 90 parts; and chloroform, 300 parts. It is claimed that Billroth has used this combination for nine years, with only one death, which occurred last summer.—*S. Practitioner.*

